# On the role of crossmodal prediction in audiovisual emotion perception

**DOI:** 10.3389/fnhum.2013.00369

**Published:** 2013-07-18

**Authors:** Sarah Jessen, Sonja A. Kotz

**Affiliations:** ^1^Research Group “Early Social Development,” Max Planck Institute for Human Cognitive and Brain SciencesLeipzig, Germany; ^2^Research Group “Subcortical Contributions to Comprehension”, Department of Neuropsychology, Max Planck Institute for Human Cognitive and Brain SciencesLeipzig, Germany; ^3^School of Psychological Sciences, University of ManchesterManchester, UK

**Keywords:** cross-modal prediction, emotion, multisensory, EEG, audiovisual

## Abstract

Humans rely on multiple sensory modalities to determine the emotional state of others. In fact, such multisensory perception may be one of the mechanisms explaining the ease and efficiency by which others' emotions are recognized. But how and when exactly do the different modalities interact? One aspect in multisensory perception that has received increasing interest in recent years is the concept of *cross-modal prediction*. In emotion perception, as in most other settings, visual information precedes the auditory information. Thereby, leading in visual information can facilitate subsequent auditory processing. While this mechanism has often been described in audiovisual speech perception, so far it has not been addressed in audiovisual emotion perception. Based on the current state of the art in (a) cross-modal prediction and (b) multisensory emotion perception research, we propose that it is essential to consider the former in order to fully understand the latter. Focusing on electroencephalographic (EEG) and magnetoencephalographic (MEG) studies, we provide a brief overview of the current research in both fields. In discussing these findings, we suggest that emotional visual information may allow more reliable predicting of auditory information compared to non-emotional visual information. In support of this hypothesis, we present a re-analysis of a previous data set that shows an inverse correlation between the N1 EEG response and the duration of visual emotional, but not non-emotional information. If the assumption that emotional content allows more reliable predicting can be corroborated in future studies, cross-modal prediction is a crucial factor in our understanding of multisensory emotion perception.

Perceiving others' emotions is an important component of everyday social interaction. We can gather such information via somebody's vocal, facial, or body expressions, and by the content of his or her speech. If the information obtained by these different modalities is congruent, a correct interpretation appears to be faster and more efficient. This becomes evident at the behavioral level, for instance, in shorter reaction times (Giard and Peronnet, 1999; Sperdin et al., 2009) and higher accuracy (Giard and Peronnet, 1999; Kreifelts et al., 2007), but also at the neural level where clear differences between unisensory and multisensory processing can be observed. An interaction between complex auditory and visual information can be seen within 100 ms (e.g., van Wassenhove et al., 2005; Stekelenburg and Vroomen, 2007) and involves a large network of brain regions ranging from early uni- and multisensory areas, such as the primary auditory and the primary visual cortex (see, e.g., Calvert et al., 1998, 1999; Ghazanfar and Schroeder, 2006) and the superior temporal gyrus (Calvert et al., 2000; Callan et al., 2003), to higher cognitive brain regions, such as the prefrontal cortex and the cingulate cortex (e.g., Laurienti et al., 2003). These data are interpreted to support the assumption of multisensory facilitation.

The fact that multisensory perception leads to facilitation is generally accepted, however, the mechanisms underlying such facilitation, especially for complex dynamic stimuli, are yet to be fully understood. One mechanism that seems to be particularly important in audiovisual perception of complex, ecologically valid information, is cross-modal prediction. In a natural context, visual information typically precedes auditory information (Chandrasekaran et al., 2009; Stekelenburg and Vroomen, 2012). Visual information leads while the auditory one is lagging behind. Thereby, visual information allows generating predictions about several aspects of a subsequent sound, such as the time of its onset and content (e.g., Arnal et al., 2009; Stekelenburg and Vroomen, 2012). Due to this preparatory information flow, the following auditory information processing is facilitated. This mechanism can be seen as an instance of predictive coding as has been discussed for sensory perception in general (see Summerfield and Egner, 2009).

The success and efficiency of cross-modal prediction is influenced by several factors, including attention, motivation, and the emotional state of the observer. Schroeder et al. (2008) for instance suggest an influence of attention on cross-modal prediction in speech perception. In the present paper, however, we will focus on a different aspect of cross-modal prediction that has largely been neglected: How does the emotional content of the perceived signal influence cross-modal prediction, or, vice versa, what role does cross-modal prediction play in the multisensory perception of emotions? Do emotions lead to a stronger prediction than comparable neutral stimuli or are emotions just another instance of complex salient information?

In the following, we will provide a short overview of recent findings on cross-modal prediction, focusing on electroencephalographic (EEG) and magnetoencephalographic (MEG) results. We will then discuss the role of affective information in cross-modal prediction before outlining necessary further steps to closer investigate this phenomenon.

## Cross-modal prediction

The most common setting, in which cross-modal prediction of complex stimuli is studied, is in audiovisual speech perception (Bernstein et al., 2008; Arnal et al., 2009, 2011). Typically, videos are presented, in which a person is uttering a single syllable. As visual information starts before a sound's onset, its influence on auditory processing can be investigated.

In EEG and MEG studies, it has been shown that the predictability of an auditory signal by visual information affects the brain's response to the auditory information within 100 ms after a sound's onset. Especially the N1 has been studied in this context (e.g., Klucharev et al., 2003; Besle et al., 2004; van Wassenhove et al., 2005), and a reduction of the N1 amplitude has been linked to facilitated processing of audiovisual speech (Besle et al., 2009). Furthermore, the more predictable visual information is, the stronger such facilitation seems to be, as suggested in MEG studies that reported a reduction in M100 latency (Arnal et al., 2009) and amplitude (Davis et al., 2008). Similar results have been obtained in EEG studies; when syllables of different predictability are presented, the syllables with the highest predictability based on visual features lead to the strongest reduction in N1/P2 latency (van Wassenhove et al., 2005).

Cross-modal prediction in complex settings has not only been investigated in speech perception, but also in the perception of other audiovisual events, such as everyday actions (e.g., Stekelenburg and Vroomen, 2007, 2012). Only if sufficiently predictive dynamic visual information is present, a reduction in the auditory N1 can be observed (Stekelenburg and Vroomen, 2007).

Regarding the mechanisms underlying such cross-modal prediction, two distinct pathways have been suggested (Arnal et al., 2009). In a first, indirect pathway, information from early visual areas influences activations in auditory areas via a third, relay area such as the superior temporal sulcus (STS). In a second, direct pathway, a cortico-cortical connection between early visual and early auditory areas is posited without the involvement of any additional area. Interestingly, these two pathways seem to cover different aspects of prediction; while the direct pathway is involved in generating predictions regarding the onset of an auditory stimulus, the indirect pathway rather predicts auditory information at the content-level, for instance, which syllable or sound will be uttered (Arnal et al., 2009). Evidence for a distinction between two pathways also arises from EEG data (Klucharev et al., 2003; Stekelenburg and de Gelder, 2004): while the N1 is assumed to be modulated by predictability of physical stimulus parameters, the P2 seems to be sensitive to the content or the semantic features of the signal (Stekelenburg and Vroomen, 2012).

In recent years, neural oscillations as a crucial mechanism underlying cross-modal prediction have come into focus (e.g., Doesburg et al., 2008; Schroeder et al., 2008; Senkowski et al., 2008; Arnal et al., 2011; Thorne et al., 2011). While the analysis of event-related potentials offers a straight-forward and reliable way to investigate brain responses closely time-locked to a specific event, the analysis of oscillatory activity provides a way to analyze changes in the EEG data with more flexible timing. Furthermore, oscillatory brain activity has been suggested as a potential mechanism to mediate the influence of one brain area onto another (Buzsaki and Draguhn, 2004). Such a mechanism may, for instance, underlie cross-modal prediction, where information from one sensory area affects the activity in a different sensory area (Kayser et al., 2008; Schroeder et al., 2008; Lakatos et al., 2009). In the case of audiovisual prediction, visual information, processed in primary visual areas, thereby has the capacity to prepare auditory areas for incoming auditory information. However, such an operation takes time (Schroeder et al., 2008), and it is therefore essential that visual information precedes the auditory one. Further, it has to provide some information about the upcoming auditory stimulus, such as an expected onset and, preferably, more detailed specification of a sound.

In summary, cross-modal prediction has been extensively studied in audiovisual speech perception and also in the perception of lower-level audiovisual stimuli. Along with an increasing interest in neural oscillations and their function(s) in recent years, new approaches and possibilities to investigate its underlying mechanisms have been developed. However, the role of cross-modal prediction in emotion perception has received hardly any attention. In the following, we will outline what is known regarding the role of emotions in cross-modal predictions.

## Emotions and cross-modal prediction

Emotion perception is a case that involves cross-modal prediction. Cross-modal prediction likely contributes to the ease and efficiency with which others' emotions are recognized. One question that arises is whether emotion perception is just one case of cross-modal prediction among others, or whether it differs substantially from cases of non-emotional cross-modal prediction.

Numerous recent studies have investigated the combined perception of emotions from different modalities (e.g., de Gelder et al., 1999; Pourtois et al., 2000, 2002; for a recent review, see Klasen et al., 2012). Emotional faces, bodies, and voices influence each other at various processing stages.

First brain responses to a mismatch between facial and vocal expressions (de Gelder et al., 1999; Pourtois et al., 2000) or also between body and facial expressions (Meeren et al., 2005) can be observed around 100 ms after stimulus onset. Interactions of matching emotional faces and voices are typically observed slightly later, between 200 and 300 ms (Paulmann et al., 2009), though some studies also report interaction effects in the range of the N1 (Jessen and Kotz, 2011). Besides these early effects, interactions between different modalities can be observed at later processing stages, presumably in limbic areas and higher association cortices (Pourtois et al., 2002; Chen et al., 2010).

However, while the processing of multisensory emotional information has been amply investigated, only recently the dynamic temporal development of the perceived stimuli has come into focus. Classically, most studies used static facial expressions paired with (by its very nature) dynamic vocal expressions (e.g., de Gelder et al., 1999; Pourtois et al., 2000).

While this allows for investigating several aspects of emotion perception under controlled conditions, it is a strong simplification compared to a dynamic multisensory environment. In a natural setting, emotional information usually obeys the same patterns as outlined above: visual information precedes the auditory one. We see an angry face, see a mouth opening, see a breath-intake before we actually hear an outcry or an angry exclamation.

One aspect of such natural emotion perception that cannot be investigated using static stimulus material is the role of prediction in emotion perception. If auditory and visual onsets occur at the same time, we cannot investigate the influence of preceding visual information on the subsequent auditory one. However, two aspects of these studies using static facial expression render them particularly interesting and relevant in the present case.

First, several studies introduced a delay between the onset of a picture and a voice onset in order to differentiate between brain responses to the visual onset and brain responses to the auditory onset (de Gelder et al., 1999; Pourtois et al., 2000, 2002). At the same time, however, such a delay introduces visual, albeit static, information, which allows for the generation of predictions. At which level these predictions can be made depends on the precise experimental setup. While some studies chose a variable delay (de Gelder et al., 1999; Pourtois et al., 2000), allowing for predictions only at the content, but not at the temporal level, others presented auditory information at a fixed delay, which allows for predictions both at the temporal and at a content level (Pourtois et al., 2002). In either case, one can conceive of the results as investigating the influence of static emotional information on subsequent matching or mismatching auditory information.

Second, most studies used a mismatch paradigm, that is, a face and a voice were either of different emotions or one modality was emotional while the other was neutral (de Gelder et al., 1999; Pourtois et al., 2000, 2002). These mismatch settings were then contrasted to matching stimuli, were a face and a voice conveyed the same emotion (or both did not show any emotional information, in a neutral case). While probably not intended by the researchers, such a design may reduce predictive validity to a rather large degree; after the first number of trials, the participant learns that a given facial expression may be followed either by the same or by a different emotion with equal probability. Conscious predictions cannot be made, neither at the content (emotional) level, nor at a more physical level based on facial features. Hence, visual information provides only limited information about subsequent auditory information. Therefore, data obtained from these studies informs us about multisensory emotion processing under conditions, in which predictive capacities are reduced. Note, however, that it is unclear to what extent one experimental session can reduce the predictions generated by facial expressions, or rather, how much of these predictions are automatic (either innate or due to high familiarity) so that they cannot be overwritten by a few trials, in which they are violated. In fact, the violation responses observed in these studies show that predictions about an upcoming sound are retained to a certain degree. However, some modulation of prediction does seem to take place, as for instance a mismatch negativity can be observed for matching face—voice pairing preceded by a number of mismatching pairings (de Gelder et al., 1999).

The results of these studies are inconsistent with respect to the influence visual information has on auditory information processing. While some report larger N1 responses for matching compared to non-matching face—voice pairings (Pourtois et al., 2000), others do not find differences in the N1 (Pourtois et al., 2002). Instead, they report later differences between matching and non-matching face—voice pairings, for instance in the P2b (Pourtois et al., 2002).

A different approach to investigate the face—voice interaction has been to present emotional facial expressions either alone or combined with matching vocal information (Paulmann et al., 2009). In this study, the onset of visual and auditory information was synchronized, thereby excluding any visual prediction before the sound onset. In such a setting, first effects of emotional information were observed in the P2, showing larger amplitudes for angry compared to neutral stimuli. While the use of matching stimuli presented in either a uni- or a multisensory way provides a promising design to investigate cross-modal prediction, the lack of any audiovisual delay prevents us from drawing any specific conclusions regarding predictive mechanisms.

Overall, visual emotional information does seem to influence auditory processing at a very early stage. However, studies investigating this influence in a natural setting are largely missing.

In two recent EEG-studies, we investigated the interaction between emotional body and voice information by means of video material in order to overcome some of the limitations of previous studies (Jessen and Kotz, 2011; Jessen et al., 2012). Videos, in which actors expressed different emotional states with or without matching vocal expressions were presented. The emotional states “anger” and “fear” were depicted via body-expressions as well as short vocalizations (e.g., “ah”). Furthermore, we included a non-emotional control condition (“neutral”), in which the actor performed a movement that did not express any specific emotion and uttered the same vocalization with a neutral tone of voice. The delay between visual and auditory onsets was different for each stimulus, as the timing of the original recording of the videos was not manipulated. Hence, the vocalization occurred with a variable delay after the actor had started to move. In both studies, we observed smaller N1 amplitudes for emotional compared to neutral stimuli, as well as for audiovisual compared to unisensory auditory stimuli, irrespective of the emotional content. The amplitude reduction for audiovisual stimuli resembles that observed by Stekelenburg and Vroomen (2007) for non-emotional stimuli, supporting the notion that the observed effect can be attributed to predictive visual information. However, we did not find an interaction with emotional content.

**Figure 1:**
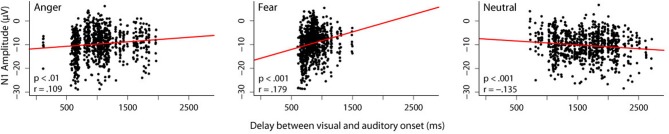
**Correlation between audiovisual delay and N1 amplitude.** In one of our studies (Jessen et al., 2012), we presented 24 participants with videos, in which different emotions were expressed by body and vocal expressions simultaneously. The delay between the visual and the auditory onset was different for each stimulus. In order to investigate the influence that a different amount of visual information has on the subsequent auditory processing, we correlated the length of the audiovisual delay with the N1 amplitude separately for each emotion. Trials in which the N1 amplitude differed more than 3 standard deviations from the mean were excluded from further analysis. Dots represent individual trials. A linear mixed model including the random factor subject and the fixed factors emotion and delay reveals a significant interaction between the fixed factors [*F*_(1, 2408)_ = 33.43, *p* < 0.0001]. It can be seen that for both emotions, an inverse relation between N1 amplitude and delay exists: the longer the delay, the smaller the N1 amplitude [anger: *F*_(1, 805)_ = 10.98, *p* < 0.001; fear: *F*_(1, 773)_ = 32.50, *p* < 0.0001]. The reverse pattern occurs in the neutral condition; here, longer delays correspond to larger N1 amplitudes [*F*_(1, 784)_ = 17.19, *p* < 0.0001].

While we did not manipulate predictive validity of the visual information in these studies, we were still interested in whether the amount of available visual information influences auditory processing. We therefore correlated the length of the audiovisual delay for each stimulus with the N1 amplitude in response to that stimulus obtained in the audiovisual condition of the experiment reported in Jessen et al. (2012) (Figure 1).

We found a positive correlation for both emotion conditions, that is, the longer the delay between visual and auditory onset, the *smaller* the amplitude of the subsequent N1. The opposite pattern was observed in the neutral condition; the longer the delay, the *larger* the N1 amplitude.

As outlined above, reduced N1 amplitudes in cross-modal predictive settings have commonly been interpreted as increased (temporal) prediction. If we assume that a longer stretch of visual information allows for a stronger prediction, this increase in prediction can explain the reduction in N1 amplitude observed with increasing visual information for emotional stimuli. However, this pattern does not seem to hold for non-emotional stimuli. When the duration of visual information increases, the amplitude of the N1 also increases. Hence, only in the case of emotional stimuli, an increase in visual information seems to correspond to an increase in visual predictability.

Interestingly, this is the case although neutral stimuli, on average, have a longer audiovisual delay (mean delay for stimuli presented in the audiovisual condition: anger: 1032 ms, fear: 863 ms, neutral: 1629 ms), and thus more visual information is available. Therefore, emotional content rather than pure amount of information seems to drive the observed correlation.

Support for the idea that emotional information may have an influence on cross-modal prediction also comes from priming research. The affective content of a prime strongly influences target effects (Carroll and Young, 2005), leading to differences in activation as evidenced by several EEG studies (e.g., Schirmer et al., 2002; Werheid et al., 2005). Schirmer et al. (2002), for instance, observed smaller N400 amplitudes in response to words that matched a preceding prime in contrast to words that violated the prediction. Also, for facial expressions, a decreased ERP response in frontal areas within 200 ms has been observed in response to primed as compared to non-primed emotion expressions (Werheid et al., 2005).

However, priming studies strongly differ from real multisensory interactions. Visual and auditory information are presented subsequently rather than simultaneously, and typically, visual and auditory stimuli do not originate from the same event. Priming research therefore only allows for investigating prediction at the content level, at which for instance the perception of an angry face primes the perception of an angry voice. It does not allow investigating temporal prediction as no natural temporal relation between visual and auditory information is present.

Neither our study referenced above (Jessen et al., 2012) nor the mentioned priming studies were thus designed to explicitly investigate the influence of affective information on cross-modal prediction in naturalistic settings. Hence, the reported data just offer a glimpse into this field. Nevertheless, they highlight the potential role cross-modal prediction may play in the multisensory perception of emotions. We believe that this role may be essential for our understanding of emotion perception, and in the following suggest several approaches suited to illuminate this role.

## Future directions

Different aspects of multisensory emotion perception need to be further investigated in order to understand the role of cross-modal prediction in this context. First, it is essential to establish the influence that emotional content has on cross-modal prediction, especially in contrast to other complex and salient information. Second, it will be necessary to investigate, which aspects of cross-modal prediction are influenced by emotional content. And finally, it is essential to consider how much or how little emotional information is sufficient to influence such predictions. We will take a closer look at all three propositions in the following.

### Affective influence on cross-modal prediction

First, it is necessary to investigate the degree to which affective content influences prediction. The correlation analysis reported above suggests that visual emotions seem to have some influence on subsequent auditory processing, but further studies are clearly needed.

In order to investigate this aspect, it is crucial to use appropriate stimulus material. Most importantly, such stimulus material has to be dynamic in order to allow for the investigation of temporal as well as content-level predictions. Only dynamic material can cover temporal as well as content predictions and, at the same time, retain the natural temporal relation between visual and auditory onsets. While the use of videos has become increasingly popular in recent years in fMRI studies (e.g., Kreifelts et al., 2007; Pichon et al., 2009; Robins et al., 2009), most EEG (and MEG) studies still rely on static material. One reason for this is probably the very advantage of EEG over fMRI, namely its high temporal resolution. While this allows for close tracking of the time course of information processing, it is also vulnerable to confounds arising from the processing of the preceding visual information. However, this problem can be countered by choosing well-suited control conditions (such as comparably complex and moving non-emotional stimuli). Furthermore, it will be helpful to not exclusively rely on ERP data, but to broaden the analysis to include neural oscillations that can be analyzed in ways less dependent on fixed event onsets (e.g., induced activity, see for instance Tallon-Baudry and Bertrand, 1999). Of particular interest in this context would be the influence emotional visual information has on the phase of oscillatory activity in auditory areas, as well as the relation between low- and high-frequency oscillations. Is, for instance, auditory processing influenced by the phase of the oscillatory activity during visual presentation?

Furthermore, it is necessary to tease apart cross-modal prediction from other forms of multisensory interaction that most likely occur in multisensory emotion perception. Here, it will be essential to manipulate the predictability of the preceding visual information, either at the content level (by for instance using different intensities of emotion expression) or at a temporal level (by providing more or less visual information, see below).

Finally, another important factor may be the role that different types of visual stimuli play, such as facial in comparison to body expressions. Both are visual sources, naturally co-occurring with auditory information, and therefore both can potentially predict auditory information. However, they differ in that facial expressions are more closely linked to vocal utterances. Body expressions, in contrast, may provide more coarse information about emotional states, essential at larger distances. Hence, while facial expressions seem the most obvious candidate, body expressions are not be forgotten (in fact, the correlation reported above shows brain data in response to body—voice pairings, Jessen et al., 2012).

Insight from these different approaches will allow us to get a general appreciation of how cross-modal prediction influences multisensory emotion perception.

### Different pathways

At a more specific level, one essential question is which aspect of cross-modal prediction can be influenced by emotional content.

One aspect that is highly relevant in this context is the notion of different pathways as outlined by Arnal et al. (2009). For cross-modal emotional prediction, at least three different levels of prediction become relevant. Predictions may occur at a simple, physical level, comparable to any other stimulus: by the movement of face and body, we can predict when an auditory event onset will occur. This prediction would correspond to the direct pathways posited by Arnal et al. (2009). This direct pathway seems to be involved in cross-modal prediction irrespective of emotional content. Emotions may render temporal predictions possibly even more reliable, as emotional facial expressions are very common, well-rehearsed stimuli and hence may allow for a more precise prediction of the onset in comparison to less frequent stimuli. However, the emotional content itself most likely plays only a minor role in the generation of temporal predictions.

Secondly, predictions may occur at the sound level. Based on the shape of the mouth (and to a certain degree other facial features), predictions can be made regarding the following utterance, be it a word, an interjection, or just a vocalization such as laughter. This type of prediction is specific to complex stimuli, for which the production of a sound can be observed visually, for instance in speech production and actions. When this is not the case, for example, if the button on a radio is pushed, we can predict the sound onset, but not the type of sound we will hear.

For this second type of predictions, emotions are expected to play a more important role, as the content of the vocalization is closely tied to the emotion expressed. Still, they not only predict emotional aspects, but also properties of the upcoming sound that are not mainly related to its emotional quality. Hence predictions specifically related to the affective content are rather a byproduct of general predicting sound features. Nevertheless, quickly determining emotional aspects is essential for fast and efficient emotion processing, and based on this necessity, affective content of the visual signal may lead to a prioritized content processing for sound information.

A third type of prediction is closely related to the prediction of a sound; with respect to cross-modal emotional prediction, we cannot only predict whether an “ah” or and “oh” will occur (as in speech perception), but also whether this “ah” will be uttered in an angry or fearful tone of voice. We can thus predict the emotional content. Both of these latter types of prediction invoke an indirect pathway (Arnal et al., 2009). However, while content prediction can occur in several settings, emotion prediction is specific to human face-to-face interaction.

This last type of predictions, emotion prediction proper, is devoted exclusively to predicting the emotional content of an upcoming signal. Hence, the strongest influence of emotional content is expected to occur at this level.

Nevertheless, in order to better understand cross-modal emotion prediction, it will be necessary to further disentangle the relation between these two types of indirect predictions (i.e., the prediction of speech content such as “ah” and the prediction of emotional content from the tone of voice).

### Duration of visual information

Another important aspect is the amount of visual information necessary to generate reliable predictions. It has been shown that the delay between the onset of mouth movement and the onset of speech sound typically varies between 100 and 300 ms (Chandrasekaran et al., 2009). Accordingly, most studies using speech stimuli use an audiovisual delay within that time range (Besle et al., 2004; Stekelenburg and Vroomen, 2007; Arnal et al., 2009). The same holds true for the perception of actions (Stekelenburg and Vroomen, 2007). However, the question arises as to how much delay is actually *necessary* to allow for cross-modal prediction to occur. Stekelenburg and Vroomen (2007), who used speech stimuli with an auditory delay of 160–200 ms as well as action stimuli with an auditory delay of 280–320 ms observed stronger N1 suppression effects for action compared to speech stimuli. They suggested that this difference may be due to the longer stretch of visual information preceding a sound onset. Somewhat shorter optimal delays have been observed using simpler stimulus material and/or more invasive recording. In human EEG, an audiovisual lag of 30 to 75 ms has been found to reliably elicit a phase reset in auditory cortex (Thorne et al., 2011). A similar time window has been found in a study of local field potential in the auditory cortex of macaque monkeys; the strongest modulation by preceding visual information was observed for a delay between 20 and 80 ms (Kayser et al., 2008).

Hence, providing more visual information may (at least up to some point) allow for a better prediction formation. At the same time, if affective information enhances cross-modal prediction, emotional content may reduce the length of required visual information. Determining the necessary temporal constraints can therefore provide crucial insight onto the effect of emotional information on multisensory information processing.

In summary, we suggest that in order to fully understand multisensory emotion perception, it is essential to take into account the role of cross-modal prediction. It will therefore be necessary to bring together approaches and findings from two flourishing fields that have so far been largely kept separate: cross-modal prediction and emotion perception. Only if we understand the role of prediction, we will be able to fully understand multisensory emotion perception.

### Conflict of interest statement

The authors declare that the research was conducted in the absence of any commercial or financial relationships that could be construed as a potential conflict of interest.
